# Accurate gingival segmentation from 3D images with artificial intelligence: an animal pilot study

**DOI:** 10.1186/s40510-023-00465-4

**Published:** 2023-05-01

**Authors:** Min Yang, Chenshuang Li, Wen Yang, Chider Chen, Chun-Hsi Chung, Nipul Tanna, Zhong Zheng

**Affiliations:** 1 Department of Orthodontics, School of Dental Medicine, University of Pennsylvania, 240 S 40Th St., Philadelphia, PA 19104 USA; 2 The Webb Schools, Claremont, CA 91711 USA; 3 Department of Oral and Maxillofacial Surgery and Pharmacology, School of Dental Medicine, University of Pennsylvania, Philadelphia, PA 19104 USA; 4 Center of Innovation and Precision Dentistry, School of Dental Medicine, School of Engineering and Applied Sciences, University of Pennsylvania, Philadelphia, PA 19104 USA; 5 David Geffen School of Medicine, University of California, Los Angeles, 675 Charles E. Young Drive, South, MRL 2641A, Los Angeles, CA 90095 USA; 6 School of Dentistry, University of California, Los Angeles, 675 Charles E. Young Drive, South, MRL 2641A, Los Angeles, CA 90095 USA

**Keywords:** Artificial intelligence (AI), Cone beam computed tomography (CBCT), Intra-oral scan, Gingiva, Segmentation, Dental informatics

## Abstract

**Background:**

Gingival phenotype plays an important role in dental diagnosis and treatment planning. Traditionally, determining the gingival phenotype is done by manual probing of the gingival soft tissues, an invasive and time-consuming procedure. This study aims to evaluate the feasibility and accuracy of an alternatively novel, non-invasive technology based on the precise 3-dimension (3D) soft tissue reconstruction from intraoral scanning and cone beam computed tomography (CBCT) to predict the gingival biotype.

**Methods:**

As a proof-of-concept, Yorkshire pig mandibles were scanned, and the CBCT data were fed into a deep-learning model to reconstruct the teeth and surrounding bone structure in 3D. By overlaying the CBCT scan with the intraoral scans, an accurate superposition was created and used for virtual measurements of the soft tissue thickness. Meanwhile, gingival thicknesses were also measured by a periodontal probe and digital caliper on the buccal and lingual sides at 3 mm apical to the gingival margin of the posterior teeth and compared with the virtual assessment at the same location. The data obtained from virtual and clinical measurements were compared by Wilcoxon matched-pairs signed-rank analysis, while their correlation was determined by Pearson’s *r* value. The Mann–Whitney *U* test was used for intergroup comparisons of the amount of difference.

**Results:**

Among 108 investigated locations, the clinical and virtual measurements are strongly positively correlated (*r* = 0.9656, *P* < 0.0001), and only clinically insignificant differences (0.066 ± 0.223 mm) were observed between the two assessments. There is no difference in the agreement between the virtual and clinical measurements on sexually matured samples (0.087 ± 0.240 mm) and pre-pubertal samples (0.033 ± 0.195 mm). Noticeably, there is a greater agreement between the virtual and clinical measurements at the buccal sites (0.019 ± 0.233 mm) than at the lingual sites (0.116 ± 0.215 mm).

**Conclusion:**

In summary, the artificial intelligence-based virtual measurement proposed in this work provides an innovative technique potentially for accurately measuring soft tissue thickness using clinical routine 3D imaging systems, which will aid clinicians in generating a more comprehensive diagnosis with less invasive procedures and, in turn, optimize the treatment plans with more predictable outcomes.

**Supplementary Information:**

The online version contains supplementary material available at 10.1186/s40510-023-00465-4.

## Introduction

Appropriate orthodontic therapies can benefit periodontal health by correcting pathological tooth migration and reducing intraosseous defects or furcation lesions [1, 2], as well as by enhancing periodontal bone regenerative outcomes via activating and stimulating the periodontal ligament [3]. However, hard and soft tissue defects, such as dehiscence, fenestration, and recession, may also be induced by orthodontic treatments [4, 5]. When considering the situation in terms of periodontal pathology, active periodontal inflammation would cause alveolar bone resorption and tooth root damage during orthodontic treatment [6], which has been haunting orthodontists recently since more and more orthodontic patients with periodontal problems are seen in the clinic, partially due to the increase of adults seeking orthodontic treatment [7]. Therefore, patients’ periodontal conditions must be closely monitored and controlled before and during the orthodontic treatment. Unfortunately, neither patients nor dental professionals are fully aware of the potential risk, etiology, and prevention strategies for periodontal complications related to orthodontic treatments [8]. Furthermore, the majority of current clinical evaluations are still limited to the hard tissues [9–11], despite the fact that about 20–25% of patients develop gingival recession 2 to 5 years after orthodontic treatment [4].

In the official proceedings from the *2018 Classification of Periodontal and Peri-Implant Diseases and Conditions* [12], a new term, *periodontal phenotype*, was adopted to describe the combination of both bone morpho-type (thickness of the bony plate) and gingival phenotype [three-dimensional (3D) gingival volume]. Accumulating studies have demonstrated that gingival phenotypes respond differently to inflammation, restorative, trauma, and parafunctional habits [13]. For instance, surgical and restorative treatments post a higher risk of dehiscence, fenestration, and recession in patients with a thin periodontal phenotype, which can be attributed to the relatively inadequate soft tissue [13]. A thin gingival phenotype is associated with the compromised blood supply to the underlying bone [14]. It may explain why tooth extraction and gingival inflammation lead to more severe alveolar resorption in patients with a thin periodontal phenotype [15] and possibly explain why the success rate of periodontal surgical procedures in those patients is also lower [16]. Moreover, the buccolingual alveolar and gingival thickness are essential concerns during orthodontic treatment [17, 18]. For example, when orthodontic forces that move the dentition outside of the alveolar housing, such as those introduced by improper arch expansion, were applied to a tooth in the zone of thin periodontal phenotype, a higher incidence of gingival recession, as well as bony dehiscence, was noticed [19]. On the contrary, a thick gingival phenotype is associated with increased stability of orthodontic outcomes and reduced periodontal complications in patients, particularly in terms of gingival recession/attachment loss [20]. Furthermore, the American Academy of Periodontology recently embarked on a Best Evidence Consensus Statement to emphasize that the periodontal phenotype critically impacts the outcome and stability of restorative, periodontal, and orthodontic treatments [21], highlighting the importance of gingival soft tissue diagnosis in clinical evaluation and treatment planning.

The current clinical methods of soft tissue thickness evaluation are conducted by subjective visual inspection, horizontal transmucosal bone sounding (*aka.* transgingival probing) [22], visual assessment of probe transparency through the gingival sulcus [23], and the use of non-ionizing ultrasonography [24]. Unfortunately, all these procedures require significant chair side time, while some involve invasive procedures that significantly increase patients’ discomfort [22]. Previously, researchers and clinicians attempted to assess the gingival thickness from clinically well-adapted intraoral scan and cone beam computed tomography (CBCT) images [25]. In this strategy, an STL file from the intraoral scan is manually superimposed onto the corresponding CBCT-based digital imaging and communications in machine (DICOM) file to assess the gingival thickness [25]. However, non-invasive measurement of the soft tissue thickness requires an accurate reconstruction of the teeth and bones underneath the soft tissues. Thus, the manual superimposition procedure requires tremendous human resources and training, markedly reducing its feasibility in clinics. More importantly, since the traditional CBCT post-processing methods rarely distinguish the boundary between hard and soft tissues accurately, the interclass correlation between the gingival thicknesses measured by the endodontic spreader and those obtained via the digital evaluation was only 0.79–0.87 [25].

Given the requirement for further improving the accuracy and reproducibility of the digital measurement of gingival thickness and to reduce human workload, we propose a novel, non-invasive method to assess soft tissue thickness by establishing an AI segmentation workflow for soft tissue thickness measurement using CBCT and intraoral scans. As a proof-of-concept study, the off-the-shelf deep-learning model is first recruited to segment teeth and bone from CBCT images of Yorkshire pig mandibles, which is then superimposed onto the intraoral scan of the pig semi-automatically. To evaluate the accuracy of this methodology, the gingival thickness obtained from the AI virtual measurements will be compared with those assessed by horizontal transmucosal bone sounding, the current clinical best practice [26]. Our hypothesis of the current study is that the trained AI models utilized in the current work are mature enough to be applied across subjects without retraining; in addition, a good agreement can be achieved between virtual and clinical measurements of soft tissue thickness.

## Materials and methods

### Study design

Swine is one of the major animal species used in dental and medical research because they share similar anatomic and physiologic characteristics with humans [27]. Particularly, Yorkshire pig heads were selected in this study due to their similar bone density, gingival thickness, and head size compared to humans [27]. CBCT and intraoral scan images were obtained under routine clinical settings. As the control method, the horizontal transmucosal bone sounding with a periodontal probe was used to measure the mandibular gingival thickness. Then, the digital models were processed using artificial intelligence (AI) platforms to accurately distinguish soft tissue from embedded hard tissue and ensure the virtual probes assessed the same locations as the clinical measurement. Finally, the gingival thicknesses quantified virtually and clinically were compared statistically to determine the validity of the novel proposed method in this study.

### Sample selection

In this study, only discarded Yorkshire pig heads were collected from research labs at the University of Pennsylvania. Thus, the Penn Institutional Animal Care & Use Committee (IACUC) Office of Animal Welfare determined that a specific IACUC approval is unnecessary. Based on the inclusive and exclusive criteria listed below, a total of seven Yorkshire pig heads were used as study samples, including four sexually matured pigs (weight 30 kg, around 6–7 months old) and three pre-pubertal pigs (weight 10 kg, approximately 1–2 months old) [27, 28] to represent different age groups of patients in orthodontic clinics.

Inclusion criteria:

1. posterior teeth were present at the mandible bilaterally;
2. intact mandible;
3. no gross gingival tissue defect.

Exclusion criteria:

1. significant deformation of the hard or soft tissue in the mandible;
2. any intraoral hard or soft tissue lesion.

### Data collection

**Fig. 1:**
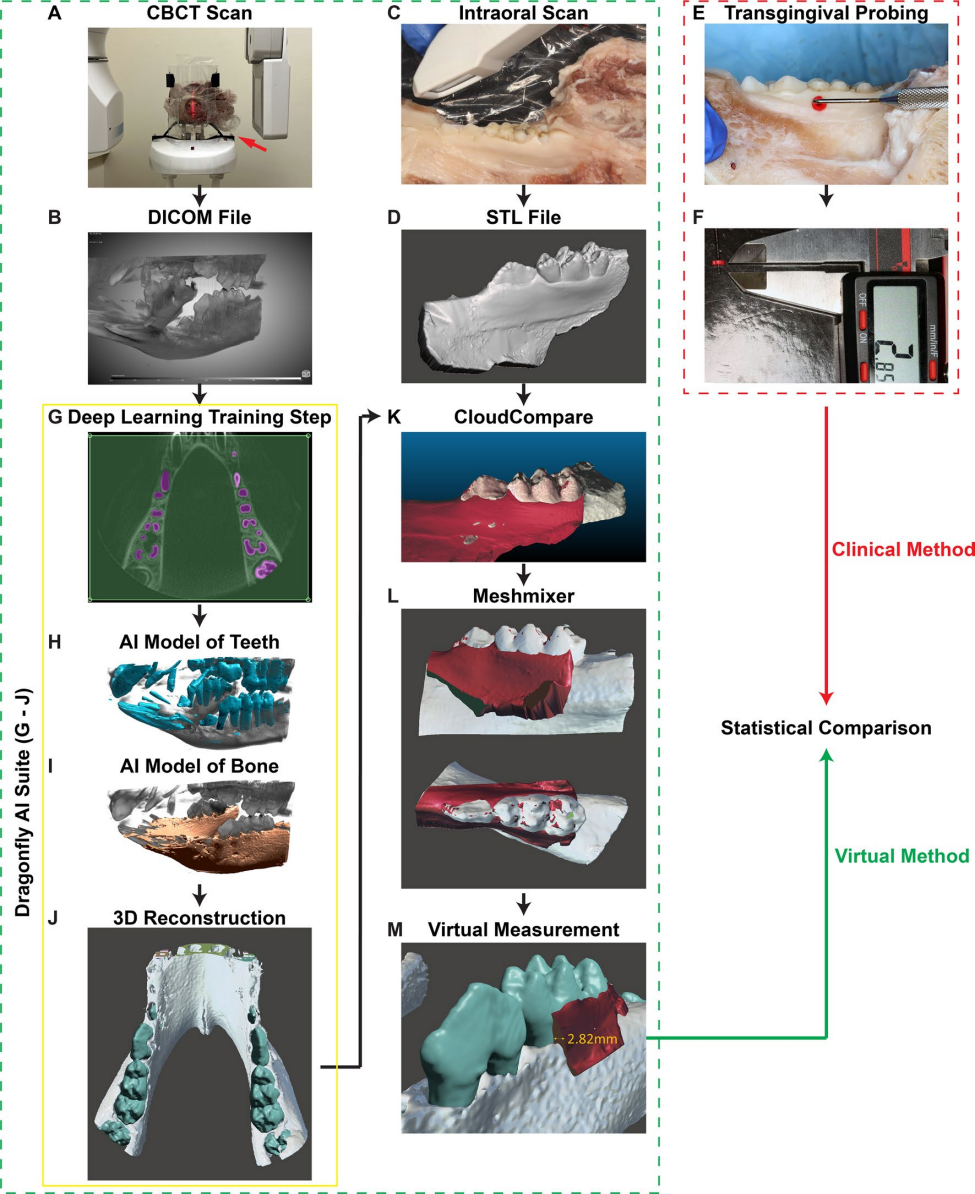
The flowchart of data collection and imaging processing of the current study. **A**, **B** CBCT scans of the pig head. The red arrow points to the 3D-printed frame, which was used to maintain the pig head position while taking the CBCT scans. **C**, **D** Intraoral scans of the pig mandibular arch. **E**, **F** Transgingival probing of the mandibular posterior teeth. **G**–**J** The AI models training process in the Dragonfly AI suite. **K** Superposition of the intraoral scans and the AI-reconstructed 3D models was performed using CloudCompare. **L**, **M** After alignment, the soft tissue thickness was measured in AutoDesk Meshmixer

The Yorkshire pig head was fixed on the headrest frame of a Planmeca ProMax^®^ 3D machine (PLANMECA USA INC, IL, USA) by a 3D-printed frame (Fig. 1A) to maintain its position during the CBCT scanning. CBCT scanning was carried out at 90 kVp and 14 mAs with a voxel size of 0.2 mm. Then, an 80 mm × 80 mm × 80 mm region of interest (ROI) containing the mandible was extracted as a DICOM file (Fig. 1B).

After CBCT imaging, the mandible of the Yorkshire pig head was dissected. An iTero intraoral scanner (Element^®^2, AZ, USA) was used to capture images of the teeth and gingival tissue of the mandibular arch (Fig. 1C). The image was then saved in STL format (Fig. 1D).

Next, the buccal and lingual gingival thicknesses were assessed at 3 mm apical to the zenith of the mid-facial and mid-lingual gingival margin at an angle perpendicular to the long axis of the posterior teeth. Briefly, a UNC 15 probe (HuFriedy Group, IL, USA) was inserted perpendicularly to the long axis of the axial root plane (Fig. 1E). When tactile resistance was encountered, indicating full-thickness penetration of the gingival tissue, the rubber stopper was passively positioned over the gingival surface. The resultant distance between the tip of the periodontal probe and the internal border of the rubber stopper was measured using a digital caliper (Fig. 1F).

### Image processing

A commercially available, well-developed software platform, Dragonfly AI suite (Version 2021.1; Object Research Systems, Montréal, Canada), was used for virtual bone and teeth segmentation. This platform was chosen because Dragonfly’s Deep Learning solution bundles with pre-built and pre-trained neural networks, implementing powerful solutions, such as U-Net [29], and its bone analysis module has been used to achieve animal and human bone segmentation quickly, accurately, and confidently [30]. In the current study, for bone segmentation, bone was manually marked on five axial-sliced images from a single sexually matured pig to span the length of the bone in the mandibular region, while seven axial-sliced images from the same pig were labeled for teeth segmentation (Fig. 1G). The settings were settled per Dragonfly’s recommendations based on similar segmentation tasks [31]. Particularly, a semantic segmentation Deep Learning model was employed with the setting of patch size: 32, stride ratio: 0.25, initial filter count: 64, Deep Learning layers number: 4, batch size: 32, epochs number: 100, loss function: OrsDiceLoss, optimization algorithm: Adadelta, and parameters count: 5,440,418. Both U-Net [32] models [teeth (Fig. 1H) and bone (Fig. 1I)] achieved a training score above 0.99, indicating convergence of the model parameters against the training inputs [31] and suggesting no further refinement is needed. Next, all slices containing teeth and bone images were used in the validation process, in which the output images of the AI models were visually assessed by overlaying the outputs against the original CBCT images. Then, the two successfully trained AI models were universally applied to all CBCT data sets obtained in this study for reconstructing 3D models that distinguished teeth and bones (Fig. 1J). These AI-segmented 3D models were exported from Dragonfly as.stl files, respectively.

Superposition of the intraoral scans and the AI-segmented 3D models was performed using CloudCompare [33] semi-automatically. Firstly, the intraoral scans and the AI-reconstructed 3D models were aligned by selected reference points to allow automatic alignment. Then, the alignment is fine-tuned manually until total visual overlap is achieved (Fig. 1K). Finally, the soft tissue thickness was measured in a 3D visualization environment, AutoDesk Meshmixer (CA, USA) (Fig. 1L). Here, the intraoral scans were sectioned at the locations where gingival thicknesses were clinically probed to expose the soft tissue (Additional file 1: Video 1). A virtual probe was then used to measure the soft tissue thickness consistent with the manual probe measurement locations (Fig. 1M).

### Statistical analysis

The previous study suggested that at least 34 measurements per clinical assessment are necessary to detect a clinically significant difference between virtual and clinical soft tissue thickness measurements [25]. Therefore, 56 sites for buccal measurements and 52 sites for lingual measurements were conducted in the current study. Among these 108 investigated sites, 43 sites were from pre-perpetual pigs and 65 sites were from sexually matured pigs.

Statistical analyses were performed with GraphPad Prism (version 8.2.1, San Diego, CA, USA). As the Shapiro–Wilk normality test revealed that not all data followed a normal distribution, data are presented as raw data overlapped with quartiles. At the same time, the mean values were also provided. The data obtained from virtual and clinical measurements were compared by Wilcoxon matched-pairs signed-rank analysis, while their correlation was determined by Pearson’s *r* value. For intergroup comparisons of the amount of difference, the Mann–Whitney *U* test was used. *P* < 0.05 was considered a statistically significant difference.

## Results

**Fig. 2:**
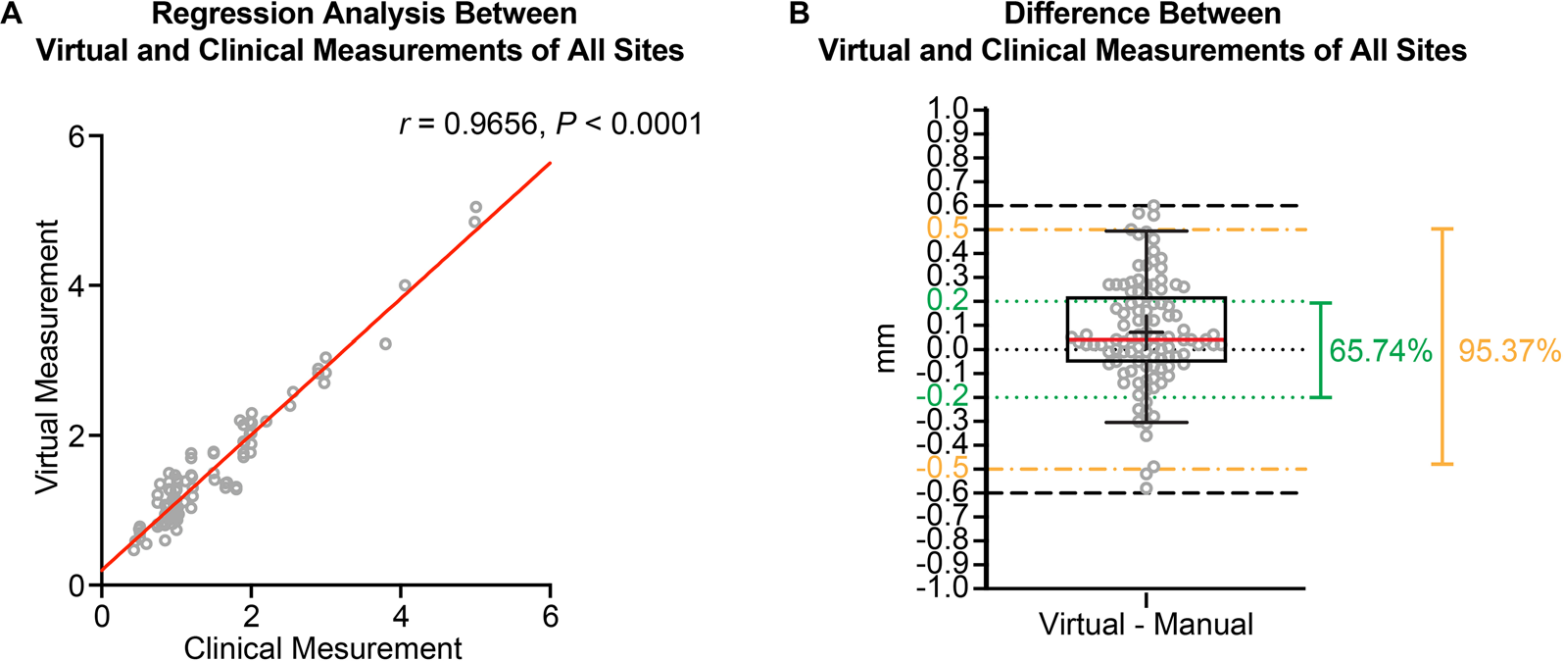
The difference between virtual and clinical probing results for all tested sites. **A** The correlation and regression results of virtual and clinical measurements. **B** The exact difference in the probing depth between virtual and clinical measurements. *N* = 108. Green dotted lines: the levels of − 0.20 mm and 0.20 mm; yellow dash-dotted lines: the levels of − 0.50 mm and 0.50 mm; black dashed lines: the levels of − 0.60 mm and 0.60 mm. The box plot represents the 5th percentile, 25th percentile, median (50th percentile, solid red line), 75th percentile, 95th percentile, and mean (black cross), respectively

When comparing the virtual and clinical measurements for all the probing sites, a correlation Pearson *r* value of 0.9656 (with a 95% confidence interval from 0.9500 to 0.9764) was achieved (Fig. 2A). The difference between the virtual and clinical measurements was 0.066 ± 0.223 mm (median 0.040 mm, range from − 0.580 to 0.600 mm, *P* = 0.0021), with 65.74% of the sites having a difference within ± 0.2 mm and 95.37% of the sites having a difference within ± 0.5 mm (Fig. 2B).

**Fig. 3:**
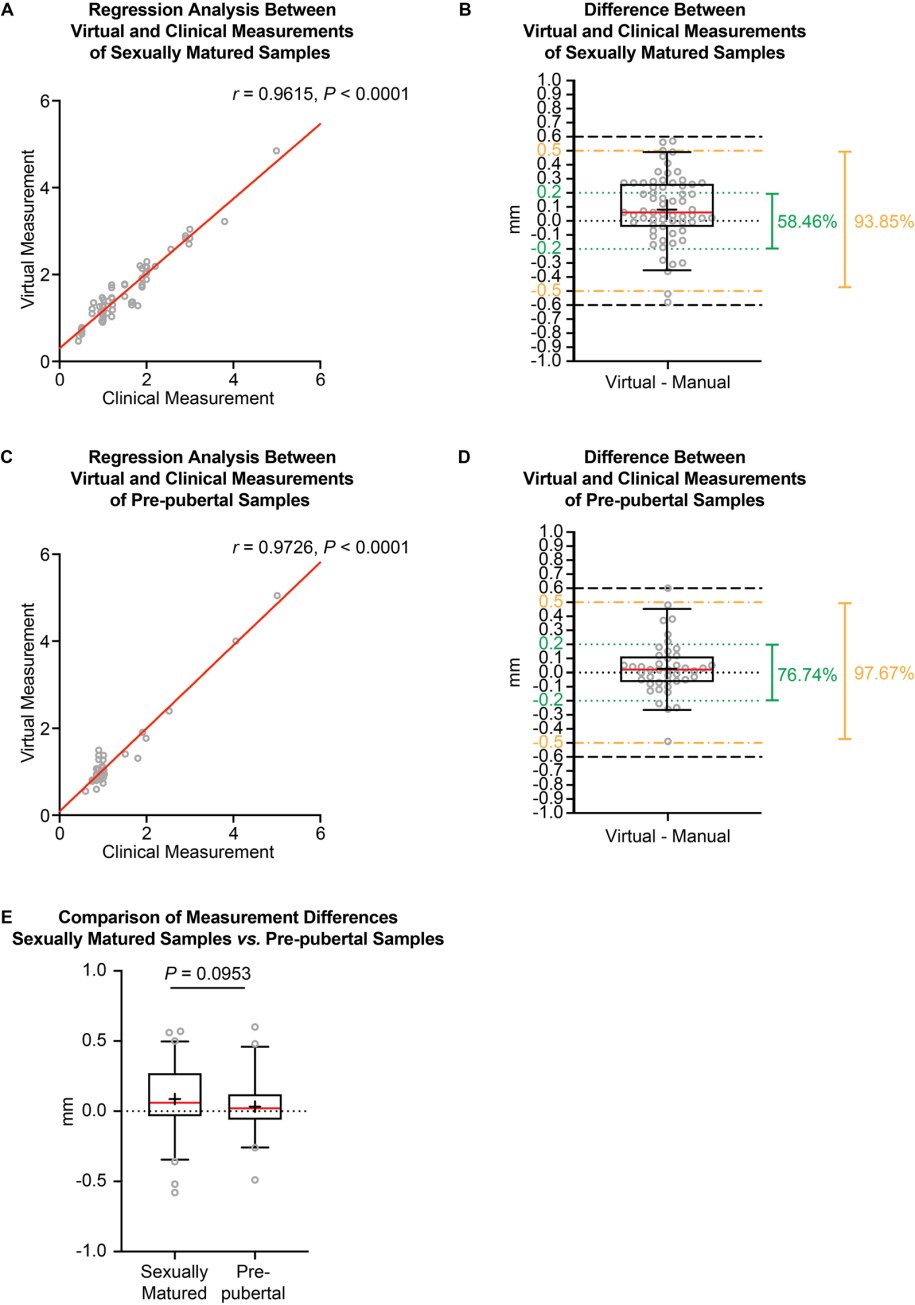
The difference between virtual and clinical probing results of sexually matured samples versus pre-pubertal samples. **A** The correlation and regression results of virtual and clinical measurements on sexually matured samples. **B** The exact difference in probing depth between virtual and clinical measurements on sexually matured samples. **C** The correlation and regression results of virtual and clinical measurements on pre-pubertal samples. **D** The exact difference of the probing depth between virtual and clinical methods on measurements on pre-pubertal samples. **E** The comparison between sexually matured samples and pre-pubertal samples for the exact difference of the probing depth between virtual and clinical measurements. The Mann–Whitney *U* test was used. *N* = 65 for sexually matured samples, *N* = 43 for pre-pubertal samples. Green dotted lines: the levels of − 0.20 mm and 0.20 mm; yellow dash-dotted lines: the levels of − 0.50 mm and 0.50 mm; black dashed lines: the levels of − 0.60 mm and 0.60 mm. The box plot represents the 5th percentile, 25th percentile, median (50th percentile, solid red line), 75th percentile, 95th percentile, and mean (black cross), respectively

Since the AI models were trained with the CBCT images from a sexually matured pig and applied to the samples from both sexually matured and pre-pubertal groups, a comparison between the two groups was conducted. As shown in Fig. 3, the correlation Pearson *r* value between virtual and clinical measurements on sexually matured samples was 0.9615 (with a 95% confidence interval from 0.9375 to 0.9764) (Fig. 3A). The difference between the virtual and clinical measurements on the sexually matured samples is 0.087 ± 0.240 mm (median 0.060 mm, range from − 0.580 to 0.590 mm, *P* = 0.0025), while 58.46% of the sites had a difference within ± 0.2 mm and 93.85% of the sites had a difference within ± 0.5 mm (Fig. 3B). For the pre-pubertal samples, the correlation Pearson *r* value between virtual and clinical measurements was 0.9726 (with a 95% confidence interval from 0.9496 to 0.9851) (Fig. 3C). The difference between the virtual and clinical measurements on the pre-pubertal samples was 0.033 ± 0.195 mm (median 0.020 mm, range from − 0.490 to 0.600 mm, *P* = 0.4183), with 76.74% of the sites having a difference within ± 0.2 mm and 97.67% of the sites having a difference within ± 0.5 mm (Fig. 3D). Moreover, no statistically significant difference was found between the sexually matured and pre-pubertal groups regarding the discrepancy between the virtual and clinical measurements (Fig. 3E).

**Fig. 4:**
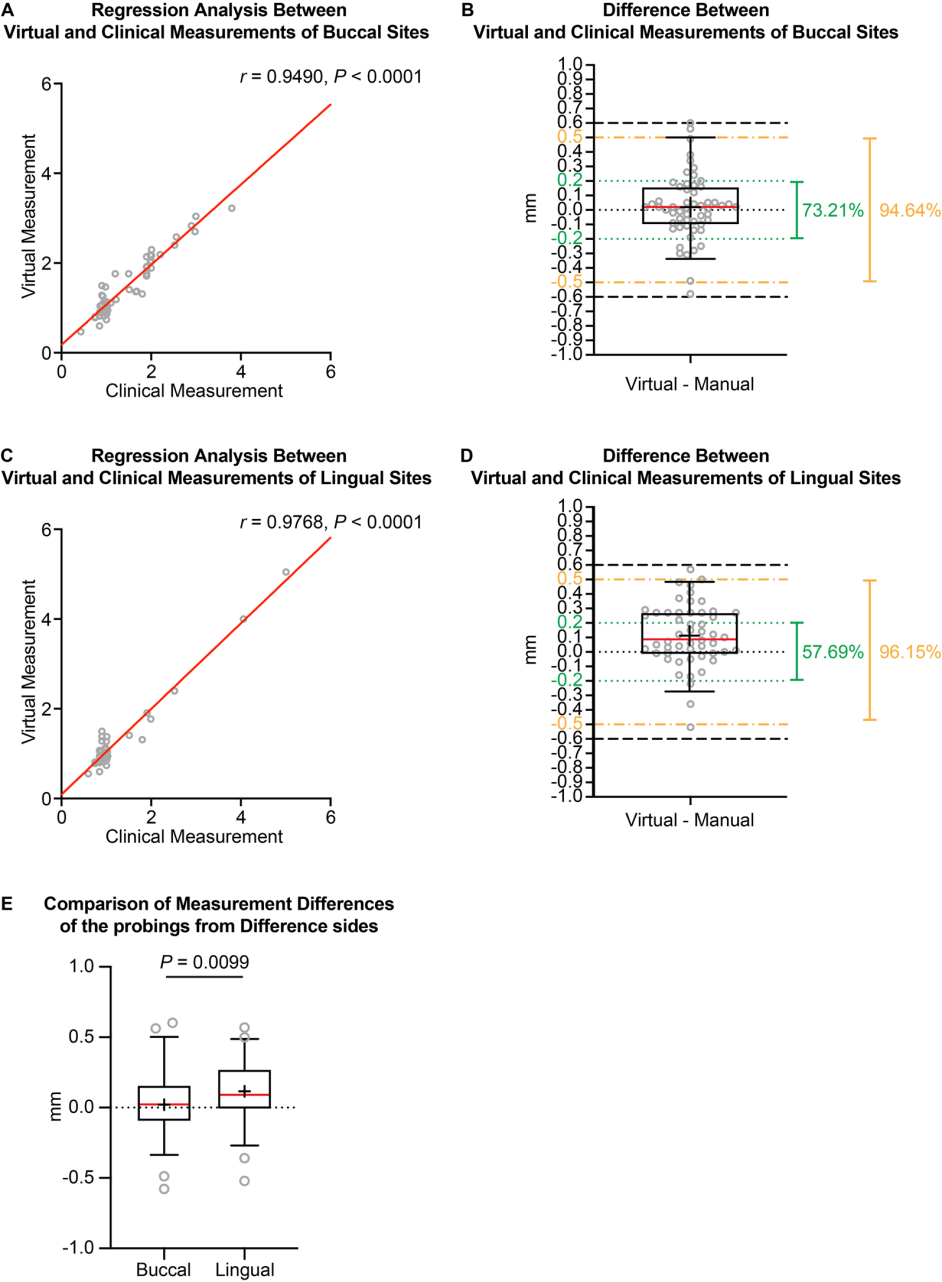
The difference between virtual and clinical probing results of buccal and lingual sides. **A** The correlation and regression results of virtual and clinical measurements of buccal probing sites. **B** The exact difference in probing depth between virtual and clinical measurements of buccal probing sites. **C** The correlation and regression results of virtual and clinical measurements of lingual probing sites. **D** The exact difference of the probing depth between virtual and clinical measurements of lingual probing sites. **E** The comparison between buccal and lingual sites for the exact difference in the probing depth between virtual and clinical measurements. The Mann–Whitney *U* test was used. *N* = 56 for buccal sites, *N* = 52 for lingual sites. Green dotted lines: the levels of − 0.20 mm and 0.20 mm; yellow dash-dotted lines: the levels of − 0.50 mm and 0.50 mm; black dashed lines: the levels of − 0.60 mm and 0.60 mm. The box plot represents the 5th percentile, 25th percentile, median (50th percentile, solid red line), 75th percentile, 95th percentile, and mean (black cross), respectively

Because the previous study showed differences in virtual probing accuracy between buccal and lingual probing sites [25], buccal and lingual probing results were also compared in the current study. The correlation Pearson *r* value between virtual and clinical measurements at the buccal sites was 0.9490 (with a 95% confidence interval from 0.9141 to 0.9699) (Fig. 4A). The difference between the virtual and clinical measurements of buccal sites is 0.019 ± 0.223 mm (median 0.020 mm, range from − 0.580 to 0.600 mm, *P* = 0.5787), with 73.21% of the sites having a difference within ± 0.2 mm and 94.64% of the sites having a difference within ± 0.5 mm (Fig. 4B). Meanwhile, for the lingual sites, the correlation Pearson *r* value between virtual and clinical measurements was 0.9768 (with a 95% confidence interval from 0.9597 to 0.9867) (Fig. 4C). The difference between the virtual and clinical measurements of lingual sites was 0.116 ± 0.215 mm (median 0.090 mm, range from − 0.520 to 0.570 mm, *P* = 0.002), with 57.69% of the sites having difference within ± 0.2 mm and 96.15% of the sites having difference within ± 0.5 mm (Fig. 4D). A statistically significant difference was detected between the buccal and lingual sites regarding the discrepancy between the virtual and clinical measurements (Fig. 4E).

## Discussion

In 2015, Ronneberger et al. presented a Convolutional Neural Network (CNN) named U-Net for medical image segmentation [32]. The proposed architecture utilizes data augmentation to drastically reduce the number of manually marked input training images, thus reducing the time and cost of model training [32]. Since then, the U-Net architecture has also been applied to dental CBCT datasets with promising results. Compared to the standard threshold-setting method for hard tissue reconstruction, recent development in AI image segmentation promises to reconstruct the teeth and bones from CBCT images with higher accuracy and less human intervention. For instance, Lee et al. reported validation and test precision of over 90% using a modified version of the U-Net architecture [34]. However, to the best of our knowledge, the current study is the first to develop an AI-based method to measure gingival thickness by the digital superimposition of STL and DICOM files and evaluate its reliability and reproducibility.

As mentioned above, all the soft tissue thickness values measured by the virtual method show excellent agreement with the clinical method. In addition, the Pearson *r* values in the current study are higher than those documented in the previous studies [25, 35]. Notably, more than 95% of the probing sites have a difference within 0.5 mm (the minimal clinically significant difference [25, 35]) between virtual and clinical measurements, highlighting the reliability of this AI-based virtual measurement in precision imaging analysis.

The excellent correlation between virtual and manual measurements also supports the applicability of the U-Net algorithm for dental CBCT image segmentation. It is worth noting that even a small number of training images (as used in the current study) can build up models capable of accurate segmentation for the entire CBCT dataset. In the current study, despite the different dentition intraorally and the different number of tooth buds in the alveolar bone [36], teeth and alveolar bone segmentations can be achieved in the images of both sexually matured and pre-pubertal pigs with the Dragonfly’s Deep Learning solution which bundles with pre-built and pre-trained neural networks. These data suggest that the proposed methodology has the potential to be universally applied to the samples with different types of dentitions without affecting the accuracy.

Excitingly, our study showed that the current method resulted in an excellent correlation between virtual and manual measurements in both buccal and lingual sites, which is different from the previous study when an AI procedure was not utilized [25]. When comparing the exact difference in probing depth, buccal sites have better accuracy than the lingual sites (0.019 ± 0.223 mm vs. 0.116 ± 0.215 mm). Still, the discrepancy between the virtual and clinical measurements on both sides is much smaller in the current study than previous study (0.08 mm for buccal sites and 0.25 mm for lingual sites) [25].

### Limitation of our method

Despite the high accuracy of the current protocol, it must be mentioned that the current methodology is established on the swine model. Furthermore, as the dental arch of the miniature pig is longer than that of a human [27], the iTero scanner could not generate a whole arch scan file. Thus, the intraoral scan images of the partial arch were used in the current study. In addition, only the mandibular arch was tested in the current study.

It is worth noting that genomic diversity and a variety of pathological conditions and treatments, such as large restoration, periapical lesions, and root canal treatment, will significantly increase the difficulty and complexity of AI-based segmentation in human patients. Moreover, the heterogeneity of machinery and technical settings of image collections should also be seriously considered when refining the AI models to achieve a broadly applicable, clinically meaningful algorithm for bone and teeth segmentation. Thus, a more rigorous model training/refining approach with large feeding data from multiple centers would be required to validate the accuracy of the model in clinical applications. No doubt, further studies are necessary to apply and verify this work in human images with both maxillary and mandibular full arch scans with a wide, preferred international collaboration among craniofacial, orthodontic, and periodontal health providers and imaging and data experts.

In addition, as a proof-of-concept study of the AI algorithm for soft tissue thickness assessment, the current workflow still requires human inputs for CBCT data normalization and alignment between CBCT and intraoral scan data. Although the current feasibility study showed promising results, an automated CBCT preprocessing protocol, such as the one described by Lee [34], could be developed. Furthermore, automated mesh alignment tools could be developed to align the AI-reconstructed bone-and-teeth model to the intraoral scan model. Lastly, a feature recognition tool should be developed to automatically probe the soft tissue thicknesses at prescribed locations.

## Conclusion

In summary, the methodology proposed in this work provides an innovative, AI-based technique for a non-invasive and accurate measurement of soft tissue thickness using clinical routine 3D imaging systems. This method holds great potential in aiding clinicians in generating a more comprehensive diagnosis and, in turn, optimizing treatment plans with more predictable outcomes in multiple aspects of periodontics and orthodontics. In addition, the availability of soft-tissue thicknesses via non-invasive methods would also allow clinicians to present the current periodontal soft tissue conditions to patients in a concise yet quantitative manner, such that patients’ expectations regarding the predicted surgical or orthodontic treatment outcomes can be better managed.

## Supplementary Information

**Additional file 1. Video 1.** The demography of the 3D model which was set up in the current study.

## Data Availability

All data generated or analyzed during this study are included in this published article.
